# Selective KRAS G12C inhibitors in non-small cell lung cancer: chemistry, concurrent pathway alterations, and clinical outcomes

**DOI:** 10.1038/s41698-021-00237-5

**Published:** 2021-11-29

**Authors:** Gabriela Palma, Faisal Khurshid, Kevin Lu, Brian Woodward, Hatim Husain

**Affiliations:** University of California San Diego, La Jolla, CA USA

**Keywords:** Non-small-cell lung cancer, Drug development

## Abstract

Cancers harboring mutations in the Kirsten rat sarcoma homolog (KRAS) gene have been associated with poor prognosis and lack of targeted therapies. KRAS mutations occur in approximately one in four patients diagnosed with non-small cell lung cancer (NSCLC) with KRAS G12C mutations harbored at approximately 11–16%. Research into KRAS-driven tumors and analytical chemistry have borne a new class of selective small molecules against the KRAS G12C isoform. Phase II data for sotorasib (AMG510) has demonstrated a 37.1% overall response rate (ORR). Adagrasib (MRTX849) has demonstrated a 45% ORR in an early study. While single agent efficacy has been seen, initial data suggest combination approaches are an opportunity to improve outcomes. Here, we present perspectives on the initial progress in targeting KRAS G12C, examine co-mutations evident in KRAS G12C NSCLC, and comment on potential future combinatorial approaches including SHP2, SOS1, MEK, EGFR, mTOR, CDK, and checkpoint blockade which are currently being evaluated in clinical trials. As of May 28, 2021, sotorasib has achieved US FDA approval for patients with KRAS G12C mutant lung cancer after one line of a prior therapy.

## Mechanism of KRAS oncogenesis and chemistry

**Fig. 1:**
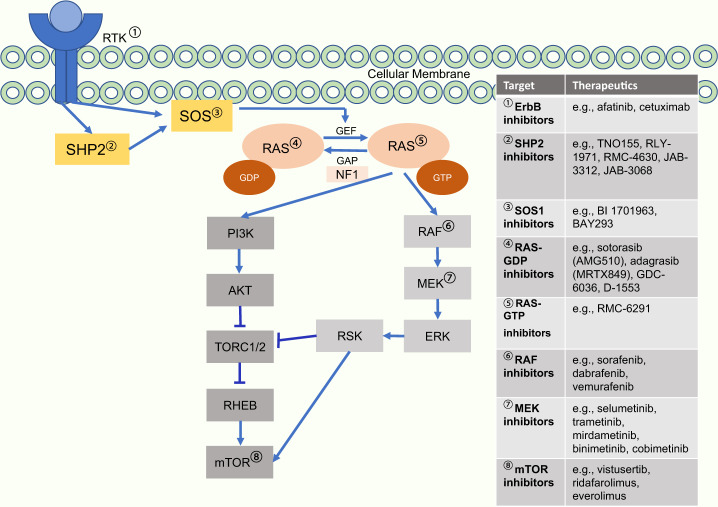
RAS/MAPK pathway downstream signaling and therapeutic targets. RAS/MAPK pathway noting therapeutic targets and some associated therapies currently being evaluated.

The KRAS protein oscillates between the inactive form of GDP-RAS and the active form of GTP-RAS^1–3^. The protein includes two major binding regions including the G domain and the C terminal membrane targeting region which, once activated through guanine nucleotide exchange factors (GEFs), have the ability to further affect many pathways including PI3K, mTOR, RAF/MEK/ERK, and other downstream cell cycle pathways^4^. To cause the shift in state of GTP-bound RAS into GDP-bound RAS, GTPase activating proteins (GAPs) hydrolyze GTP Fig. 1 leading to a reduction in cell growth seen when GTP is bound to RAS and serving as a functional control mechanism of proliferation^5^. Somatic mutations block the reversible nature of this gene, creating a constitutively bound RAS-GTP complex and resulting conformational change.

G12C mutations make up about 13% of NSCLC, 3–5% of colorectal cancers, and 1–2% of other cancer types. A genomic analysis of KRAS mutations in NSCLC showed that a G>A transition has been increasingly seen in those who have never smoked, while a G>T transversion is commonly seen in current and former smokers who are diagnosed with lung cancer^6^. In comparison, KRAS mutations are less common in lung squamous cell carcinoma (<1–5%) and small cell lung cancer (1–2%)^7–10^. Among other types of cancers, KRAS mutations appear in approximately 97% of pancreatic adenocarcinomas, 52% of colorectal cancers, and some responses have been seen in these tumor types albeit with varying consistency^3^. Within a subgroup analysis of those who are more frequently diagnosed with KRAS G12C NSCLC, both Caucasian and African American populations can be more often diagnosed than Asian populations. Interestingly, white females harbor KRAS G12C NSCLC mutations more often than their male counterparts. This trend is opposite for Asian populations, as Asian males more frequently have NSCLC G12C mutations^11^.

Cancers harboring RAS mutations have remained genomically untargetable more than 30 years after the initial discovery of the oncogene, and attempts to target KRAS directly or through downstream mechanisms have been met with limited efficacy in clinical trials. Earlier attempts to target KRAS have focused on molecules that bind directly to RAS, inhibit interaction with its activator SOS or its effector RAF, affect downstream signaling (i.e., MEK inhibitors), act on synthetically lethal pathways, and affect binding at the plasma membrane (i.e., farnesyltransferase inhibitors). Unfortunately, none of these mechanisms have resulted in approvals for these strategies in KRAS G12C mutant cancers. KRAS binds tightly with GTP (with picomolar affinity in vivo) in the main binding pocket, and its features sterically inhibit many treatments from reversing the strong binding affinity for GTP^4^. In order for RAS to maintain its functionality, the molecule requires a modification with C15 farnesyltransferase when localized in the plasma membrane^3^. This modification was the target of an active pipeline of farnesyltransferase inhibitors and yielded preclinical responses however limited responses in clinical trials^12^.

**Fig. 2:**
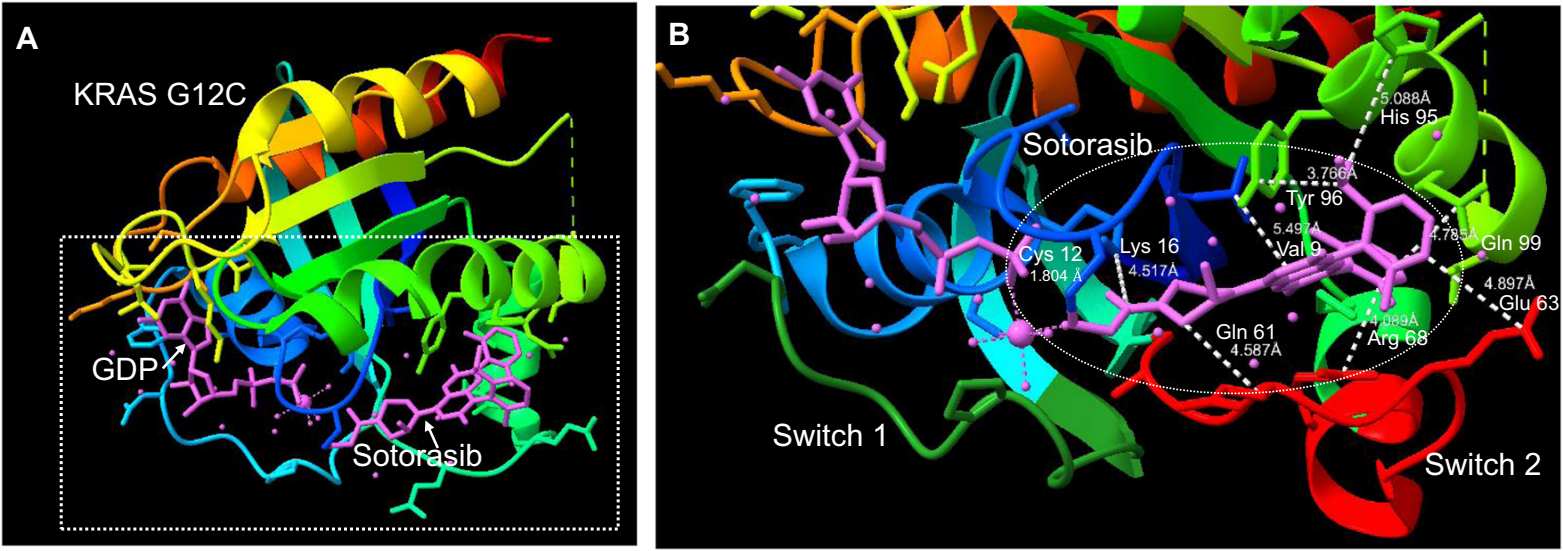
Crystalline protein structure of KRAS G12C modeled. **A** General representation of KRAS G12C protein in 3-dimensional space in relationship with bound sotorasib and GDP. The outline box represents area that is focused on in (**B**) view. **B** Distances between localized KRAS G12C side-chains to sotorasib (in pink, encircled in white) are presented. Switch pockets 1 and 2 are highlighted in dark green and red, respectively. Loci we identified included: Val9, Lys16, Gln61, Glu63, Arg68, His95, Tyr96, and Gln99. This has been modelled via UCSF ChimeraX^13,14^.

We have modeled KRAS G12C and presented areas of binding of the medicine sotorasib at the allosteric pocket under the Switch II loop region (Fig. 2) in the GDP-bound state of KRAS using ChimeraX software from UCSF^13,14^. Binding at this pocket has been found to inhibit RAS activity by blocking SOS-mediated nucleotide exchange and altering the affinity of KRAS for GDP versus GTP nucleotide. These innovative approaches demonstrated the presence of previously unknown binding pockets on the surface of RAS and provided a framework for continued efforts to develop novel RAS targeted therapies^15,16^. Small molecules bind irreversibly with high specificity to the mutant cysteine at exon 12, leading to preferential GDP binding over GTP^16^. Decreased binding affinity to the downstream RAF protein target contributed anti-tumor effects. One of the first developed mutant specific, covalent small molecule inhibitors to demonstrate proficiency in targeting KRAS G12C and locking it into a GDP-bound state was ARS-853 by Wellspring Biosciences and had robust cellular activity against KRAS G12C in the low micromolar range. Modifications were made to this small molecule inhibitor, giving rise to ARS-1620, which targeted the His95 amino acid on KRAS G12C. ARS-1620 also proved to have a higher potency and preclinical efficacy and produced a better conformational response than its predecessor^1^.

## In vitro and pre-clinical trial data demonstrates anti-tumor activity

Direct inhibition of KRAS G12C was validated by ARS-1620, but the identification of improved inhibitors suitable for clinical testing proved difficult. A key challenge was suboptimal potency owing to the small volume of the pocket occupied by ARS-1620, which offered limited avenues for additional protein-ligand interactions. The discovery that a surface groove, created by an alternative orientation of His95, could be occupied by aromatic rings and enhance interactions with the KRAS G12C protein was important to identify therapeutic vulnerabilities (Fig. 2). Sotorasib (AMG 510) emerged as the top candidate from optimization of His95 groove-binding molecules. Although portions of the AMG 510 and ARS-1620 ligands are structurally related, the His95 groove binding is a novel feature of AMG 510. Enhanced interactions improved the potency of AMG 510 as compared to ARS-1620 in a nucleotide-exchange assay with recombinant GDP-bound KRAS G12C. The kinetics of the reaction between AMG 510 and GDP-KRAS G12C were measured by mass spectrometry and exhibited a marked improvement compared to ARS-1620. Preclinical work in a variety of cancer cell lines from lung cancer and other solid tumors harboring the G12C mutation demonstrated anti-tumor response^17^.

In parallel, a structure-based drug design approach and optimization led to the discovery of adagrasib (MRTX849) as a potent, covalent KRAS G12C inhibitor. In xenografts and preclinical models, a precursor tool compound MRTX1257 showed promising results, leading to development of MRTX849^18,19^. The preclinical maximally efficacious dosage that promoted optimal tumor regression while maintaining tolerance was between 30-100 mg/kg per day. Pre-clinical mouse models (CDX and PDX) also showed a total response rate of 65% across tumors, and specifically a response rate of 75% in NSCLC models^20^. In pre-clinical models utilizing H358 and MIAPaCa-2 xenografts, evaluation of adagrasib showed that concomitant mutations (i.e., TP53, KEAP1, or STK11) did not predict against therapeutic response.

The function of KRAS and its RAF-MEK-ERK pathway depends largely on the activation of the KRAS scaffold, comprised of proteins including Src homology region 2 domain-containing phosphatase 2 (SHP2) and son of sevenless homolog 1 (SOS1). The combined interactions of SHP2 and SOS1 transition the GDP-bound state of KRAS to the GTP-bound state, rendering the protein active and allowing for proliferation. SHP2 and SOS1 have been demonstrated to serve as drug targets in controlling a mutant KRAS. In addition, SHP2 activation enables resistance mechanisms to MEK inhibitor therapy and enhances expression of signaling upstream from KRAS^21^. SOS1 has been labeled as a potential oncogenic driver, with the capability of tumorigenesis in vivo and the ability to activate both the RAS protein through a RAS-GEF (guanine nucleotide exchange factor) and Myc target genes, as well as increase phosphorylation of MEK^22^. This provided the rationale for a therapeutic approach of combining a KRAS G12C inhibitor with a MEK inhibitor to anticipate the potential resistance, and early investigations with AMG510 demonstrated preclinical efficacy. In another study evaluating SOS1 interactions in the KRAS pathway, chemical inhibition of SOS1 with BAY-293 completely suppressed the pathway in wild-type KRAS cells. In mutant-KRAS tumor cells, inhibition of SOS1 led to a 50% reduction in phospho-ERK. When combined with the KRAS G12C inhibitor, ARS-853 and BAY-293 worked together in NCI-H358 cells to produce a synergistic response^23^. Additional in vitro combination screen assays were performed to determine the possible combination therapies that may further support adagrasib and sotorasib combinatorial strategies. Possible escape mechanisms for the tumor were identified through in vitro and in vivo models and CRISPR screens and included HER family receptor tyrosine kinases, EGFR, SHP2, and mTOR/S6 pathways^19^.

Scaffold proteins can mediate therapeutic resistance mechanisms to reactivate the RAS/MAPK pathway by RTK signaling. KRAS G12C inhibitors have been characterized by adaptive resistance through re-activation of KRAS scaffold proteins, emphasizing the importance of targeting RTKs and other cooperative resistance mechanisms in potential combinations. KRAS G12C inhibition via ARS-1620 has been shown to cause temporary pathway suppression followed by RAS-MAPK rebound activation in KRAS G12C mutant lung cell lines. KRAS G12C inhibition in combination with the RTK inhibitors afatinib and erlotinib have demonstrated synergy in vitro models. The use of combination SHP2 and KRAS G12C inhibition have been shown to decrease tumor growth in in vivo models compared to monotherapy alone. SHP2 has been described to be a mediator between RTK signaling and KRAS activation. SHP2 inhibition combined with KRAS G12C inhibition decreased KRAS-GTP active conformation and MAPK pathway activation. The described benefit of SHP2 and KRAS G12C inhibition supports further investigation of combination therapy approach to prevent upregulation of upstream pathways^24^.

**Fig. 3:**
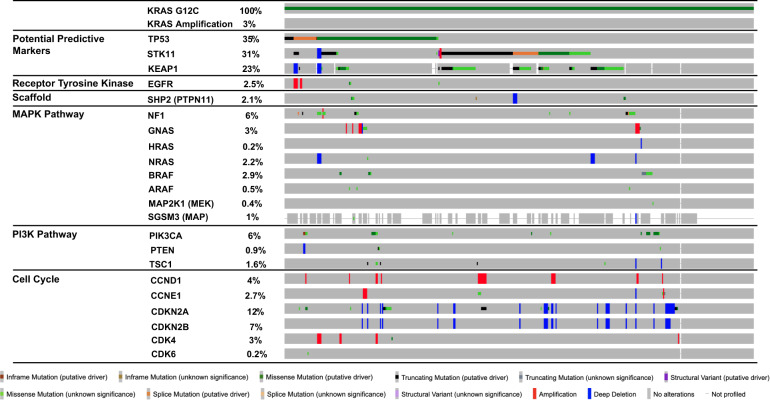
Landscape of co-alterations with KRAS G12C in Non-small Cell Lung Cancer. Co-occurring genomic alterations found in KRAS G12C mutant NSCLC patients (*n* = 560, cBioPortal.org) including those that have combinatorial strategies planned in clinical trials^25,26^.

Based on non-small cell lung cancer datasets within cBioPortal, the landscape of KRAS G12C mutated tumors includes a complex landscape of co-altered genes (Fig. 3)^25,26^. Frequently co-altered genes include TP53, STK11, KEAP1, and CDKN2A. The significance of targeting the co-altered genes is being explored in ongoing studies and biomarkers beyond genomic aberrations include RNA-based signatures and protein expression.

## Clinical trials show initial efficacy

### Sotorasib

The FDA approval of sotorasib has been a remarkable example of the speed of recent FDA approvals for clinically active medicines. The phase I investigation of sotorasib was initiated in August 2018 with the CodeBreak 100 study (NCT #03600883). Preliminary results for the phase I study presented in 2019 showed a partial response (PR) for sotorasib in 5 of 10 patients with KRAS G12C-mutant NSCLC and 4 patients achieved stable disease (SD) for 90% disease control rate by investigator review. In 18 patients with KRAS G12C mutant colorectal cancer (CRC), 13 achieved SD^27^. Approximately 66.1% of patients reported adverse events (AEs) from the treatment, which included nausea and diarrhea. 18.6% of the total enrolled patients reported one or more AEs of grade of 3 or higher that were deemed treatment related. The mean elimination half-life of the drug was determined to be 5.5 h. Dose escalation ranged from 180 mg to 960 mg, and responses were seen in all dose levels evaluated.

In 2020, updated results of 59 evaluable NSCLC patients were presented and 19/59 achieved PR (32.2% ORR) and 33/59 achieved SD (88.1% DCR). Of the 59 patients, 34 were treated at 960 mg with an ORR of 35.3% and DCR of 91.2%. In colorectal cancer, 3 of 42 patients achieved PR (ORR 7.1%) and another 28/42 achieved SD (DCR 73.8%). 28 patients with diagnosed cancers other than NSCLC or colorectal cancer were evaluated, with four reaching PR and 17/28 achieving SD. Within the NSCLC cohort, the median progression-free survival (PFS) was 6.3 months, and within the colorectal cancer cohort the PFS was 4.0 months^28^.

In a more mature Phase II cohort presented in 2021, 126 patients with previously treated NSCLC were enrolled and presented, 124 of which were response eligible by central review. Results demonstrated an ORR of 37.1%, including 43 partial responses and 3 complete responses. Disease control rate was 80.6%^29^. Median duration of response was 10.0 months and median progression-free survival was 6.8 months. This data was granted FDA priority review on February 16, 2021 and the phase 3 clinical study comparing the efficacy of sotorasib versus docetaxel for patients with KRAS G12C-mutant NSCLC is actively ongoing^30^.

An updated analysis of the 126 patient Phase II NSCLC cohort has been recently published^31^. In the updated analysis, the overall response rate remained at 37.1% although one of the partial responses converted to a complete response. The updated analysis provided the first report of overall survival for NSCLC patients treated with a selective KRAS G12C inhibitor. The median overall survival was estimated at 12.5 months, which compares favorably to historical benchmarks in this disease setting (i.e. docetaxel combined with ramucirumab had 10.5 months overall survival in the REVEL study)^32^. Subgroup analyses demonstrated consistency of benefit for both overall response and overall survival across various subgroups including prior lines of therapy and prior treatment history^33^. Phase II and Phase III development proceeded with the 960 mg dose, although the sponsor has announced they will conduct a randomized study of sotorasib 240 mg versus sotorasib 960 mg as part of a US FDA post-marketing requirement^34^.

### Adagrasib

In KRYSTAL-01 (NCT #03785249), a phase I/Ib and II trial (*n* = 79) to investigate the efficacy of adagrasib, the majority of patients were included in the dosage cohort of 600 mg taken twice daily, although doses up to 1200 mg twice daily were evaluated. In the combined Phase I/Ib and Phase II NSCLC cohorts of patients (*n* = 51) receiving 600 mg twice daily, 45% achieved PR while 51% achieved SD for a DCR of 96%. In the combined cohort of 18 patients with colorectal cancer who received 600 mg twice daily (*n* = 18), 17% achieved PR, while 78% achieved SD for a total disease control rate of 94%. In patients with other types of tumors (non-NSCLC, non-CRC) who were administered 600 mg twice daily (*n* = 6), ORR was determined to be 67% with one response unconfirmed and all patients remaining on treatment at data cutoff. The half-life of adagrasib was determined to be ~24 h. Combining all enrolled patients for safety analyses, 85% of patients enrolled in the clinical trial experienced AEs, with 30% experiencing grades 3–4, and 2% experiencing grade 5 adverse events. In the cases of fatal events, one patient experienced cardiac failure and another experienced pneumonitis^35^.

Exploratory analyses for both sotorasib and adagrasib have analyzed the impact of co-occurring alterations on efficacy. For sotorasib, an ORR of 50% was observed in 22 KRAS G12C patients with STK11 mutation and no KEAP1 mutation^29^. However, in the 13 patients with triple mutation of KRAS G12C, STK11, KEAP1, the ORR was 23%. For adagrasib, an ORR of 64% was observed in 14 KRAS G12C/STK11 co-altered patients (versus 33% in 30 KRAS G12C mutant/STK11 wild-type patients)^35^. The analysis of KEAP1 within the adagrasib STK11 subset has not been described at this time. The efficacy signals observed with STK11 co-alterations may provide insight into opportunities for evaluation in front-line NSCLC or with combination approaches. KRAS G12C inhibition may be a favorable approach in patients with concomitant STK11 mutations given findings that these patients may represent a population whom respond poorly to PD-1 therapy based on retrospective studies^36^.

## Other molecules under investigation

Several other novel compounds are currently under active evaluation to understand differences in terms of potency and on target resistance (Table 1)^28,37–45^. A modification to the ARS-1620 molecule has been brought forward as JNJ-74699147 and entered clinical development in July 2019 by Janssen. The phase I study completed recruitment (NCT #04006301) and stopped enrollment with additional information not publicly disclosed at this time. Another KRAS G12C small molecule inhibitor, LY3499446, has been advanced by Eli Lilly but since discontinued and a new small molecule inhibitor with more selectivity is currently being evaluated as LY3537982^46^.

**Table 1:** Current Clinical Trials with G12C inhibitors in NSCLC and other tumors.

KRAS G12C—sotorasib and adagrasib				KRAS G12C—other emerging agents				KRAS scaffold (SHP2 and SOS1)		
Drug	Tumor	Combination	Trial	Drug	Tumor	Combination	Trial	MOA	Drug	Trial
Sotorasib	Solid Tumors (NSCLC & CRC Expansion)	PD-1	CodeBreak 100^28,37^ NCT03600883	GDC-6036	Solid Tumors (NSCLC & CRC Expansion)	PD-L1 EGFR mAb VEGF EGFR TKI	GO42144 NCT04449874	SHP2	TNO155^43^	NCT03114319 NCT04000529 NCT04330664 NCT04294160 NCT04699188
	Solid Tumors (NSCLC & CRC Focus)	PD-1 MEK SHP2 Pan-ErbB TKI PD-L1 EGFR mAb Chemotherapy mTOR CDK4/6	CodeBreak 101^38^ NCT04185883	LY3499446	NCT04165031 terminated				JAB-3312	NCT04121286 NCT04045496 NCT04720976
	NSCLC 2^nd^ line	Sotorasib vs docetaxel	CodeBreak 200^39^ NCT04303780	JNJ-74699157	NCT04006301 discontinued				JAB-3068	NCT03518554 NCT03565003 NCT04721223
	Solid Tumors	–	CodeBreak 105 NCT04380753	JDQ443	Solid Tumors	PD-1 SHP2	NCT04699188		RMC-4630^44^	NCT03634982 NCT03989115 NCT04185883 NCT04418661
Adagrasib	Solid Tumors (NSCLC & CRC Expansion)	PD-1 EGFR mAb Pan-ErbB TKI	KRYSTAL-01^40^ NCT03785249	D-1553	Solid Tumors (NSCLC & CRC Expansion)	–	NCT04585035		RLY-1971	NCT04252339
	Solid Tumors (NSCLC & CRC Expansion)	SHP2	KRYSTAL-02^41^ NCT04330664						BBP-398	NCT04528836
	NSCLC	PD-1	KRYSTAL-07^42^ NCT04613596						ERAS-601	NCT04670679
	NSCLC 2^nd^ line	Adagrasib vs docetaxel	KRYSTAL-12 NCT04685135						PF-07284892	NCT04800822
	CRC 2^nd^ line	Adagrasib + Cetuximab vs chemotherapy	KRYSTAL-10 NCT04793958					SOS1	BI 1701963^44^	NCT04111458 NCT04627142

Genentech is evaluating a KRAS G12C small molecule inhibitor GDC-6036 (NCT #04449874). This small molecule inhibitor has been shown to have high potency and selectivity^47^. InventisBio has developed a KRAS G12C inhibitor (D-1553), which has been positioned in NSCLC and colorectal cancer. The phase I/II is ongoing and will include evaluation of the drug as monotherapy and in combination with standard cytotoxic therapy (NCT #04585035)^48^. In the beginning of January 2021, Novartis KRAS G12C inhibitor compound JDQ443 is undergoing a phase Ib/II clinical trial as a monotherapy and in combination with either TNO155 or spartalizumab (NCT #04699188)^49^. Revolution Medicine is developing RMC-6236, a pan RAS inhibitor which may be able to target both exon 12 mutations and exon 13 mutations^4^.

In addition to the KRAS G12C molecules currently in clinical development, there are novel molecules in preclinical stage development that have additional differentiating characteristics. While all of the current clinical stage molecules bind to the inactive GDP-bound conformation of KRAS G12C, Revolution Medicines is advancing a compound, RMC-6291, that binds to the active GTP-bound conformation of KRAS G12C^50^. The compound binds to the active GTP-bound conformation via a tri-complex approach in which the molecule binds a chaperone protein and in turn complexes with the active KRAS conformation with expected IND filing in 2022^51^. Meanwhile, others including Eli Lilly are developing more potent forms of GDP-bound KRAS G12C inhibition^52^. LY3537982 is a novel KRAS G12C inhibitor that binds KRAS G12C with higher potency and target occupancy than sotorasib and adagrasib in preclinical studies and has a first-in-human trial planned for 2021.

## Pathways forward with combinatorial approaches

Clinical utility in previously untreated NSCLC and in other cancer types such as colorectal cancer will likely require greater efficacy than that seen to-date with KRAS G12C inhibitor monotherapy. Strong emphasis towards combination development is currently underway with a wide variety of combinatorial approaches currently under investigation including MEK, KRAS scaffold (SHP2, SOS1), EGFR, CDK4/6, mTOR, and cytotoxic chemotherapy (Table 1). The mechanisms described here are adapative signaling patterns that have been described preclinically after sotorasib or adagrasib exposure in tumor models (Fig. 1).

### MEK inhibition

In vitro combinations have been evaluated combining sotorasib and inhibitors of HER kinase, EGFR, SHP2, PI3K/AKT, and MEK. Investigations evaluating both single agent and combinatorial efficacy of MEK inhibitors selumetinib and trametinib have demonstrated improvements in some clinical endpoints and have demonstrated responses across KRAS isoforms. Co-occurring STK11 and KRAS mutations in mouse models show increased resistance to MEK inhibition, compared to responses in mice with concomitant KRAS and p53 mutations. The addition of a MEK inhibitor to docetaxel demonstrated improved outcome in patients with KRAS-mutant NSCLC compared to either single agent treatment, combination with erlotinib, or docetaxel alone^53^. Based on the in vitro data, MEK inhibitors with lower dosages of sotorasib demonstrated an investigative opportunity forward^4^. Combinatorial efficacy of a MEK inhibitor with chemotherapy, as well as with the addition of a G12C inhibitor are being evaluated^54^. Clinical combinations of mTOR inhibitors with MEK inhibitors yielded severe adverse effects, and have prompted preclinical trials to test the substitution of a MEK inhibitor with the KRAS G12C inhibitor ARS-1620, in combination with an mTOR inhibitor (everolimus) and an insulin-like growth factor 1 receptor (IGF-1R) inhibitor (linsitinib)^55^. Both sotorasib and adagrasib are being evaluated in early clinical studies in combination with the MEK inhibitor trametinib.

### KRAS Scaffold: SHP2 and SOS1

Other targets in the KRAS scaffold, namely SOS1 and SHP2, are also being actively investigated for combination potential. SHP2 (PTPN11) is a tyrosine phosphatase in which assists in activation of RAS through the RAS/MAPK pathway (Fig. 1). SHP2 inhibition can be efficacious in targeting for BRAF RAS-GTP cancers, inactivation of neurofibromin 1 (NF1), and RAS cancers in which an oscillation of RAS is seen. Through both knockout and inhibition of SHP2, SOS1 can be inhibited and RAS/MAPK is restricted in oncogenic proliferation^56^.

Multiple SHP2 inhibitors are being investigated in both monotherapy and combination with KRAS G12C inhibitors. Revolution Medicine and Sanofi have initiated development of RMC-4630, which is being investigated in phase I/II clinical trials as a monotherapy and in combination. Pre-clinical models have shown efficacy of RMC-4630 in combination with sotorasib^57^. A tool SHP2 inhibitor compound developed by Revolution Medicine is RMC-4550, which showed significant in vivo results when targeting KRAS G12C H358 and MIAPaCa-2 xenograft models both in terms of potency and sensitivity^56^. Relay Therapeutics has developed RLY-1971, an oral SHP2 inhibitor, and is investigating the drug as a monotherapy in a phase I clinical trial^58^. Genentech has collaborated on the development of RLY-1971 for a combination approach with KRAS G12C. Novartis is currently investigating the efficacy of TNO155 as monotherapy and in combination with G12C inhibition. Jacobio and AbbVie are jointly developing two oral small molecule SHP2 inhibitors including JAB-3068 and JAB-3312 which are being evaluated as monotherapies and in combination with a KRAS G12C inhibitor^59^.

A novel investigation into KRAS G12C therapeutic approaches has yielded a new target of the SOS1 catalytic domain that affects the EGFR/MAPK pathway. Through inhibition of SOS1, the binding of KRAS to GTP and the constitutively active state is reduced. Multiple KRAS G12 and G13 mutations are able to be inhibited. The SOS1:KRAS inhibitor BI-3046, developed by Boehringer Ingelheim, has been evaluated in vivo in NSCLC and CRC PDX models specifically in combination with trametinib (MEK inhibitor). Pre-clinical models showed that the combination of the SOS1:KRAS and MEK inhibition resulted in tumor regression. Thus far, the combination of trametinib with Boehringer Ingelheim’s SOS1:KRAS inhibitor BI 1701963 has shown both time- and dose-dependencies within MAPK pathway targets. Phase I monotherapy and combination therapy clinical trial results are expected soon, as BI 1701963 will be evaluated with both trametinib and adagrasib^60^.

### EGFR, CDK4/6, mTOR

Sotorasib and adagrasib are being evaluated with CDK inhibitors, an mTOR inhibitor, MEK inhibitors, and EGFR inhibitors^61^. When achieving maximal response from adagrasib, association was seen with ERK activity inhibition. A HER family inhibitor (afatinib), CDK4/6 inhibitor (palbociclib), and mTOR pathway inhibitor may complement the efficacy of adagrasib with further improvements in response based on adaptive signaling. Afatinib, RMC-4550, vistusertib (mTOR), everolimus (mTOR), and palbociclib combinations were evaluated in cell line and xenograft models to explore dual pathway inhibition. Each tested combination proved to significantly enhance anti-tumor activity of adagrasib compared to any individual therapy alone^19^.

The role of CDK4/6 inibitors in KRAS mutated disease has been an active area of interest. Abemaciclib, a cyclin-dependent kinase (CDK) 4/6 inhibitor was investigated in the JUNIPER clinical trials against erlotinib in patients with KRAS mutant (exon 12 or exon 13) advanced NSCLC. The outcome showed a similar median OS between the two therapies, which was statistically insignificant. The cohort treated with abemaciclib experienced a PFS of 3.6 months, an ORR of 8.9%, and a DCR of 54.4%. The cohort treated with erlotinib experienced lower values, including a PFS of 1.9 months, an ORR of 2.7%, and a DCR of 31.7%. These latter three values were statistically significant, demonstrating clinical activity of CDK4/6 inhibition within KRAS exon 12 and exon 13 NSCLC patients^62^. Ongoing work evaluates CDK4/6 inhibitors in combination with G12C selective inhibitors^19^.

### Immune checkpoint inhibitors in G12C NSCLC

Approved immunotherapies for NSCLC and/or SCLC are pembrolizumab, nivolumab, ipilimumab, atezolizumab, and durvalumab. PD-1 monoclonal antibody therapy has been approved as a first-line option for patients as either a monotherapy or in conjunction with platinum-based chemotherapy in NSCLC. Patients with KRAS G12C aberrations were included in frontline immunotherapy clinical trials including KEYNOTE-189, KEYNOTE-042, KEYNOTE-024, and CheckMate-227. Upon stratification of patients with KRAS G12C, it has been found in KEYNOTE-042 that the ORR for pembrolizumab monotherapy was 66.7%, while the ORR for chemotherapy was 23.5%. The median PFS for patients treated with pembrolizumab was 15 months, as compared to 6 months for patients treated with chemotherapy^63^. In patients with a concomitant mutation of STK11, however, the therapies had less benefit, leading to STK11 being labeled as a potentially negative predictor for immunotherapy however additional work is ongoing in this field^36^. In preclinical studies, KRAS G12C direct inhibitors were noted to upregulate a pro-inflammatory tumor microenvironment and enhance anti-tumor T cell activity. Enhancement of the immune activity led to response seen with sotorasib in CT-26 cell line models^17^. Currently, multiple trials are investigating the potential of G12C inhibitors with existing immune checkpoint inhibitors (Table 1). As anti-PD1 antibodies can have durable responses, the synergistic effects of the therapies may be important in patients with KRAS G12C-mutant NSCLC.

While clinical data for PD-1 checkpoint inhibitors and KRAS G12C combinations is highly anticipated, clinical data on the sequencing of PD-1/PD-L1 checkpoint inhibitors and sotorasib is an important area for exploration. Per recent subgroup analyses of the Phase II NSCLC cohort from CodeBreaK 100, patients appear to derive benefit after PD1/PD-L1 checkpoint inhibition, including the application of the checkpoint inhibitor within 3 months of sotorasib initiation^31^. The vast majority of patients (115 of 126) received prior PD-1/PD-L1 checkpoint inhibition and the response rate was 36.3% (vs. 37.1% in the overall population) and median overall survival was 12.0 months (versus 12.5 months in the overall population). Patients whom have had checkpoint therapy within 3 months had overall response of 34.4% and median overall survival of 11.7 months, compared to 39.7% and not evaluable in patients that had more than 3 months between checkpoint inhibition and sotorasib. These data may indicate that prior immune therapy does not meaningfully negatively impact the activity of sotorasib, suggesting a possible combination approach may be further explored given non-overlapping mechanisms of action.

### Acquired resistance mechanisms

Mechanisms of resistance from direct G12C inhibitors are currently under investigation. A study recruited repeat biopsy or ctDNA samples from 23 patients with NSCLC and seven with CRC, all treated with adagrasib^64^. 42% of this cohort experienced multiple resistance mutations following treatment, while the rest of patients experienced a single resistance mutation. Mutations identified in the switch II binding pocket of *KRAS* included R68S, H95D, H95R, and Y96C. We observed in our structural model the proximity of Val9, Lys16, Gln61, Glu63, Arg68, His95, Tyr96, and Gln99 to bound sotorasib (Fig. 2). Given the position of these amino acids, mutations may impede binding or function of sotorasib. Alterations also occurred at the cysteine residue of G12, as well as in exon 13. Patients further acquired on-target amplifications of KRAS G12C and off-target alterations of BRAF, NRAS, MEK, and gene fusions. Importantly, a histological change was observed in some patients through a proposed transformation of adenocarcinoma to squamous cell carcinoma (SCC), without any further acquired resistance mechanisms^64^. Similar histological transformations have been observed after EGFR inhibitor therapy^65^. An understanding of how one G12C inhibitor compares to another based on mechanisms of resistance with mutations in the switch II binding pocket may be important in determining the sequence of therapy and treatment of resistance.

## Future directions with G12C inhibitors

### Is there front-line potential in NSCLC?

The most important alternative for G12C selective inhibitors in therapeutic landscape remain anti-PD1 therapy in patients with lung cancer. Immunotherapy with checkpoint inhibitors remain a standard of care for front line management in KRAS mutated NSCLC. Comparing the emerging KRAS G12C clinical data in NSCLC with chemotherapy and immunotherapy in KEYNOTE-189, with the caveat that patient characteristics differ between trials, activity of the KRAS G12C inhibitors in delaying disease progression appears more robust than standard platinum doublet therapy (PFS 4.9 months) however less than pembrolizumab (PD-1 inhibitor) plus platinum doublet therapy (PFS 9.0 months). KRAS mutation-positive patients derived benefit in both PFS and OS from the combination of pembrolizumab plus chemotherapy as opposed to platinum doublet therapy alone as first-line therapy for patients with metastatic NSCLC^66^. Sotorasib is being evaluated in combination with platinum chemotherapy and in combination with PD-1 inhibitors. Data suggesting that patients with KRAS-mutant NSCLC with STK11/LKB1 co-occurring mutation may have higher responses and may have attenuated responses to immune checkpoint therapy^36^. A cohort of the adagrasib KRYSTAL-01 study is evaluating adagrasib in previously untreated KRAS G12C/STK11 co-mutated NSCLC as this disease subset has poor prognosis with chemotherapy or immunotherapy and is an area of unmet need.

### Do these molecules have dose-dependent CNS efficacy?

The central nervous system is an important space for targeted therapy drug development. It is important to note that patients with active brain metastases were excluded from both initial trials for both sotorasib and adagrasib, providing a lack of data on how the small molecule inhibitors penetrate the central nervous system. The sotorasib CodeBreak 101 has recently included an arm to specifically investigate the efficacy of monotherapy on patients with brain metastases^61^. Preclinical data of adagrasib efficacy on KRAS G12C brain metastases in a LU99Luc mouse model show a dose-dependent CNS effect with tumor regression noted^20^. Further work is necessary to confirm whether these drugs may have a neuroprotective effect^67^. As each of the inclusion criteria for both sotorasib and adagrasib did not permit patients with active brain metastases to participate, further studies are needed to assess CNS response.

### Is there an opportunity to expand the reach of molecular profiling for mutation detection?

A major challenge in navigating the most effective therapeutic path for patients involves access to molecular profiling to identify which patients may have the presence of driver and resistance mutations, as approximately 10-25% of NSCLC cases are not eligible for a biopsy or do not have adequate tissue^68,69^. Next-generation sequencing of circulating tumor DNA in plasma samples using ultra-sensitive sequencing technologies with a short turn-around time have facilitated increased detection^70^. Concordance of tissue mutations detected in plasma samples can be ~90% in late-stage lung cancer^71,72^. Serial monitoring for the re-emergence of tumor-derived mutations is being evaluated to understand progression of disease based on radiographic imaging^73^. The current clinical trials have broadened eligibility to include circulating tumor DNA for some KRAS G12C trials. Amgen and Guardant have announced plans to develop a liquid biopsy companion diagnostic for sotorasib^74^.

## Conclusion

The FDA has granted priority review for sotorasib, leading to approval in May of 2021 and availability of this compound for wide clinical use. Recent overall survival data has been favorable with an overall survival of 12.5 months including second and third line and beyond patients receiving benefit. Favorable ORR, DOR, PFS, and a favorable toxicity profile relative to current standards in the previously treated setting demonstrate high potential utility in clinical care. Ongoing work to identify the mechanisms of resistance to sotorasib and adagrasib can lead to rationale combinations of targeted therapy with second site mutations in KRAS identified as well as bypass pathways. Specifically how mechanisms of resistance to each compound may influence sequence of therapy and combinatorial strategies is an active area of ongoing exploration.
